# Enhancing Impact Energy Absorption, Flexural and Crash Performance Properties of Automotive Composite Laminates by Adjusting the Stacking Sequences Layup

**DOI:** 10.3390/polym13193404

**Published:** 2021-10-03

**Authors:** Hassan Alshahrani, Azzam Ahmed

**Affiliations:** 1 Department of Mechanical Engineering, College of Engineering, Najran University, King Abdulaziz Road, P.O. Box 1988, Najran 61441, Saudi Arabia; 2 Department of the Textile Engineering, School of Engineering and Technology Industries, Sudan University of Science and Technology, Khartoum, Sudan; azzam.ko@hotmail.com

**Keywords:** stacking sequences, flexural property, low-velocity impact damage, failure mode

## Abstract

In response to the high demand for light automotive, manufacturers are showing a vital interest in replacing heavy metallic components with composite materials that exhibit unparalleled strength-to-weight ratios and excellent properties. Unidirectional carbon/epoxy prepreg was suitable for automotive applications such as the front part of the vehicle (hood) due to its excellent crash performance. In this study, UD carbon/epoxy prepreg with 70% and 30% volume fraction of reinforcement and resin, respectively, was used to fabricate the composite laminates. The responses of different three stacking sequences of automotive composite laminates to low-velocity impact damage and flexural and crash performance properties were investigated. Three-point bending and drop-weight impact tests were carried out to determine the flexural modulus, strength, and impact damage behavior of selected materials. Optical microscopy analysis was used to identify the failure modes in the composites. Scanning electron microscopy (SEM) and C-scan non-destructive methods were utilized to explore the fractures in the composites after impact tests. Moreover, the performance index and absorbed energy of the tested structures were studied. The results showed that the flexural strength and modulus of automotive composite laminates strongly depended on the stacking sequence. The highest crash resistance was noticed in the laminate with a stacking sequence of [[0, 90, 45, −45]_2_, 0, 90]_S_. Therefore, the fabrication of a composite laminate structure enhanced by selected stacking sequences is an excellent way to improve the crash performance properties of automotive composite structures.

## 1. Introduction

Advancement in technology is pushing the automotive industry to another level to produce automobiles at low cost without compromising structural integrity, weight carrying capacity, and speed. In the modern era, the industry has settled on the ideology that composite materials exhibit unparalleled strength-to-weight ratios and excellent properties, making them the prime choice for manufacturing composite structures [1,2,3]. Composite materials are essentially made up of several materials with various chemical and physical properties. From this fusing effect, the resulting structure differs significantly compared to individual component properties. Composite materials reduce the weight of automobile structures, resulting in less fuel consumption and contributing to the increased demand for this new technology [4]. Similarly, there has been growing interest in using carbon fiber-reinforced plastic (CFRP) composites for structural parts in automotive research on the role of stacking sequences on the flexural and impact damage of the composite conducted. Carbon fibers are the primary material that are then sandwiched together with a plastic resin to make CFRP. Compared to metal alloys and metal, the resulting product has a higher strength-to-weight ratio that is less susceptible to fatigue and corrosion.

The invisible damages are dangerous in that they may increase during normal operation under unstable loading conditions, eventually causing complete damage to the product. In the real world, structures made of composite materials are more likely to encounter low-velocity impact, which can cause damage and impede the structural integrity [4,5]. In most cases, the extent of the damage will act as the determining factor in the structure’s functionality; thus, an in-depth investigation is necessary to unravel all potential shortcomings in using composite materials in the automobile industry. From the same perspective, two types of stacking sequences of the composite laminate structure were examined under a three-point bending load [6]. Ary Subagia et al. [7] investigated hybrid composite laminate flexural properties of various stacking sequences of carbon and basalt fabrics. The researchers concluded that placing the basalt fabric on the broadside improved the fiber sequence flexural modulus and strength of hybrid composite laminates. In other words, the appropriate stacking sequence of materials improves hybrid composite laminate’s mechanical properties. Several studies have investigated composite laminate’s bending properties by assessing various factors such as stacking, fiber orientation, and production conditions [8,9,10,11,12,13]. Nunes et al. [14] suggested that the flexural behavior of composites is influenced by several aspects, including laminate stacking, molding temperature, fiber orientation, and surface waviness. The experimental data in this research were validated along with those drawn from the ALGOR program (finite element program software), where a difference of up to 13% was recorded between the simulated and experimental flexural stiffness values.

In essence, damage can occur not only on the surface of the polymer composites but also beneath them by relatively minor impacts, leading to visible damages. Several studies have investigated the extent of damage on composite laminate when exposed to low-velocity impact based on stacking sequence [3,11,15,16,17,18]. To test the effects of low-velocity damage in composite laminates, Russo et al. [19] subjected film-sacked composite laminate plates to a falling weight test to analyze the damage behavior of virgin polypropylene. They concluded that recycled polyolefins had lower impact parameters. As SEM morphological studies confirmed, the impact results posited an enhancement in the interfacial adhesion to fiber breakage. Additionally, the prototypical dissipative procedure of damage improved under the testing conditions [19]. Similarly, Kwon et al. [20] compared the mechanical performance of carbon fiber-reinforced polypropylene (CF/PP) and random fiber-reinforced polypropylene and their performance against a steel fender, with the results indicating that CF/PP lamination had imperfect resin impregnation. The results alluded that the insertion of RFP led to improved resin impregnation. Nonetheless, the authors asserted that the composite be substantiated with metal alloys for physical parts. Careful selection of the reinforcement to be used is crucial and depends on the purposes and functions of several components. CFRP composites are prone to stress concentration because of their intrinsic brittleness. Even a slight static overload or accidental impact could lead to severe structural damage, such as matrix delamination or cracking [21].

According to Mahesh et al. [22], selecting composite constituents appropriately for a specific application is a demanding job for an engineer or designer. In their study, the authors explored various matrix and fiber combinations used in impact applications and documented that existing research focuses primarily on the impact behavior of CFRP around the traditional conventional stiff composites, with sacrificial structures receiving little attention when exposed to the impact. High strain rates or impact loads are almost unavoidable in composite material applications; thus, their static strength is not the only consideration, and impact behavior and energy absorption properties are also crucial [23]. In some instances, efforts to refine the in-plane mechanical properties lead to compromise in impact performance. On that note, Ali et al. [23] mentioned that efficient and secure structure design must consider intensive cognizance of the composites’ impact behavior to allow manufacturing of excellent composites with exceptional impact properties and in-plane characteristics in the future. The overall idea behind the growth of composite structures in the automotive industry is that they are expected to excel in terms of performance in environments where low-velocity impacts are prevalent [21]. Additionally, critical assessment and analysis of techniques used to improve composite structures, including interleaving, have been hampered by several constraints, such as design complexities and high tooling costs, subsequently leading to mechanical property degradation. In a similar study, Aymerich and Francesconi [24] examined the effect of stitching on the damage and structural strength of carbon/epoxy laminates exposed to low-velocity impact and reported that although both stitched and unstitched laminates exhibited similar impact responses based on force–displacement curves, force–time histories, and energy absorption properties, the stitches considerably improved the damaged resistance of the laminate. In other words, in stitched laminate, impact response is a result of competition between the stitches’ bridging action, consequently limiting delamination growth. Past studies have used several nondestructive testing (NDT) modalities, like sonic infrared imaging [25], X-ray computed tomography (CT), thermography [26,27,28], and ultrasonic and acoustic emissions, among others, to identify and detect types of damages in composite structures.

Low-velocity impact damage is one of the major issues concerning the durability of composite laminations, as it causes invisible damage on the surface. On that note, the detection of invisible damage induced by the low-velocity impact, such as delamination and matrix cracking in carbon composite laminates, has to be investigated in depth to reduce the likelihood of their occurrence. In addition, given that the manufacturing and material cost is relatively high, the automobile industry must conduct more research on this new technology for its extensive use in manufacturing. The advantages of this technology are too appealing to ignore. The demand for lightweight automobiles and a reduction in fuel consumption is on the rise. Furthermore, a high amount of energy absorption without transferring excessive deformation to the inner structure is one of the fundamental requirements of a crash-resistant structure for transportation. Therefore, this study contributes to the existing knowledge by investigating the responses of UD carbon/epoxy prepreg using three different stacking sequences ([[0, 90, 45, −45]_2_, 0, 90]_S_, [0, 90]_10_, and [[0, 90, 45, −45]_2_, [0, 90]_6_]) for low-velocity impact damage, and flexural and crash performance properties. This can improve the crash performance properties of automotive composite structures. The reason for selecting these composite lay-ups with quasi-static and unbalance stacking sequences was to provide more impact energy absorption rather than detectable delamination. However, identifying dominant damage mechanisms and locations in the selected materials by various techniques helps to validate its applicability. Consequently, this research characterizes failure and damage modes using SEM, C-scan, and optical microscopy.

## 2. Materials and Methods

### 2.1. Materials and Fabrication

**Figure 1:**
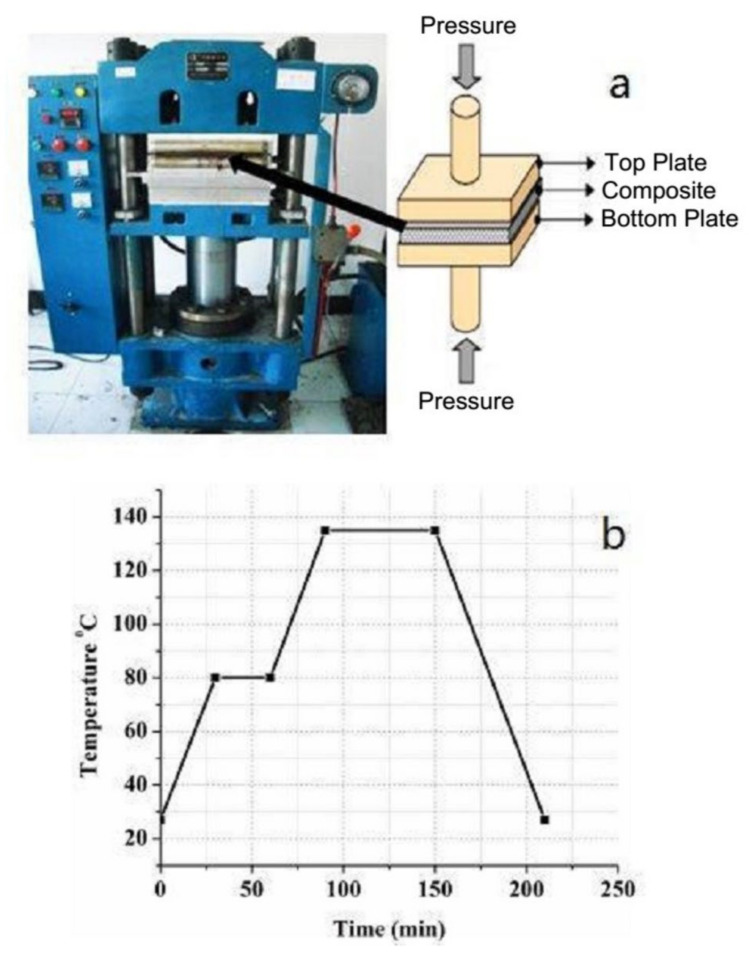
(**a**) Compression molding machine (flat-panel vulcanizer) and (**b**) the curing process.

Carbon fiber/epoxy (unidirectional prepreg, areal weight 179 g/m^2^) was supplied by Weihai Guangwei Composites Co., Ltd., Weihai, China. Table 1 shows the properties of the prepreg. Laminates consisting of three different stacking sequences were made by a compression molding process shown in Figure 1a. The processing pressure was 2 MPa, curing for 30 min at 80 °C, and post-cure for 60 min at 135 °C with a temperature rise rate (heating rate) of 1.2 °C/min (Figure 1b). The dimension of the whole sample was 320 mm × 320 mm (length × width). Table 2 presents three different stacking sequences of the composites.

### 2.2. Flexural Test

**Figure 2:**
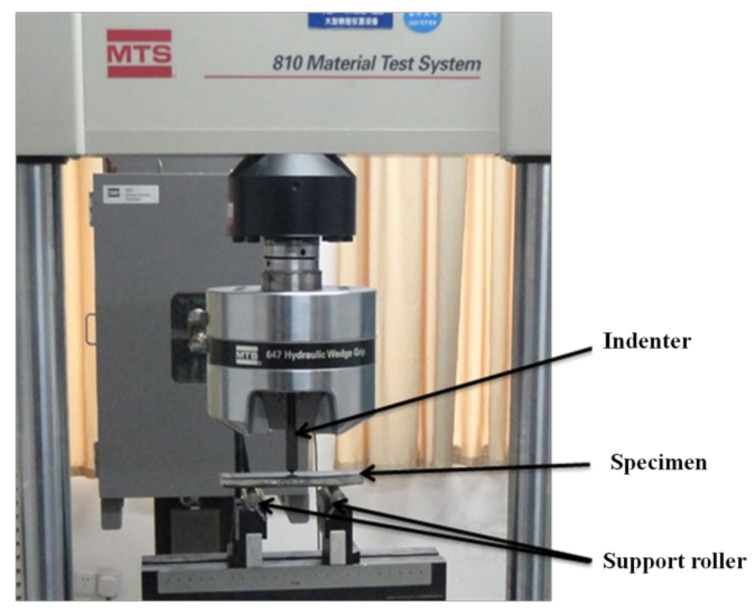
Three-point bending test using an MTS machine.

Flexural tests were carried out using a three-point bending test in accordance with ASTM D7264 [29], and at least three specimens for each laminate were tested. The tests were performed in a Material Testing System (MTS 810-647 hydraulic wedge grip, MTS Systems Corporation, Eden Prairie, USA) machine (Figure 2). The applied velocity was 2 mm/min, with an indenter diameter of 8 mm. The bending test specimens had dimensions of 170 mm in length and 30 mm in width and were all cut with a water jet cutter. Optical microscopic analysis was used to identify the failure mode in the specimen after the bending test. The flexural strength (*σ_f_*) and modulus (*E_f_*) of the composite laminate were calculated according to the following equations [9,29]:

(1) $$\sigma_{f}=\frac{3PL}{2bd^{2}}$$

(2) $$E_{f}=\frac{L^{3}m}{4bd^{3}}$$

where *L* is the support span (mm), *b* is width of the specimen (mm), *d* is depth of the specimen, *P* is the flexural load, and *m* is the initial slope of the load–displacement curve.

### 2.3. Low-Velocity Impact Test

**Figure 3:**
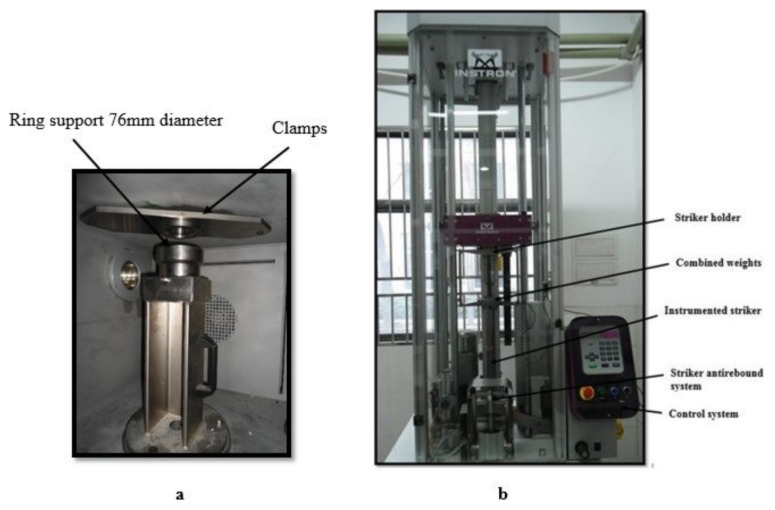
CEAST 9350 impact tester (**a**) test chamber and (**b**) device system.

The INSTRON CEAST 9350 drop tower impact tester machine (Instron Corporation, Norwood, MA, USA), as shown in Figure 3, was used to investigate the impact properties. The ASTM 7136 standard [30] was used as the baseline for testing. The testing process involved dropping a 2.277 kg hemispherical striker with a diameter of 16 mm on the subjects from different heights. The composite laminate was subjected to various energies of 5 J, 15 J, and 25 J. The incident velocity was 2.10, 3.63, and 4.69 m/s, with corresponding drop heights of 224.8, 671.8.78, and 1121.5 mm. Please note that low-velocity impact events occur in the range of 1 to 10 m/s depending on the material properties and the projectile mass [31,32]. At every impact energy level, at least three samples were tested. Following the impact test, the damage in the composite sample was inspected and analyzed using a scanning electron microscope (TM3000, HITACHI, Tokyo, Japan). Moreover, optical microscopic analysis was used to identify the failure mode in the specimen after the impact test. During an impact event, these equations were used to compute the absorbed energy from the load–displacement curves [33,34]:

(3) $$E_{ab}=\frac{mv_{0}{}^{2}}{2}-\frac{mv_{i\left(t\right)}{}^{2}}{2}$$

where $E_{ab}$ is the absorbed energy, $v_{0}$ is the initial velocity, $v_{i\left(t\right)}$ is velocity of the impactor at the time, and m is the mass of the striker.

### 2.4. Non-Destructive Test

To minimize the possibility of contamination, a noncontact ultrasonic detection system (NAUT21, Japan Probe Co., Yokohama, Japan) was used for the non-destructive test, as presented in Ref. [35]. This facility eliminates the need to apply a coupling agent to the specimen [35,36]. The detection part, with 400 kHz frequency, encompasses a top probe (pulser) and a bottom probe (receiver). The diameter of the probe is 20 mm. The operational procedure entailed placing the material under examination between the left and right clamp holders, which are adjustable depending on the sample size required, and modification of the holder height ensured the sample was kept at an equal distance from the pulser and receiver. Then, to obtain the fundamental healthy material pulse, the pulser and receiver devices were moved above and below the undamaged region. Subsequently, the amplitude, frequency, measurement range, and scanning speed testing parameters were perfectly set. The sample was then scanned to obtain the final C-scan image.

## 3. Results and Discussion

### 3.1. Flexural Strength and Modulus

**Figure 4:**
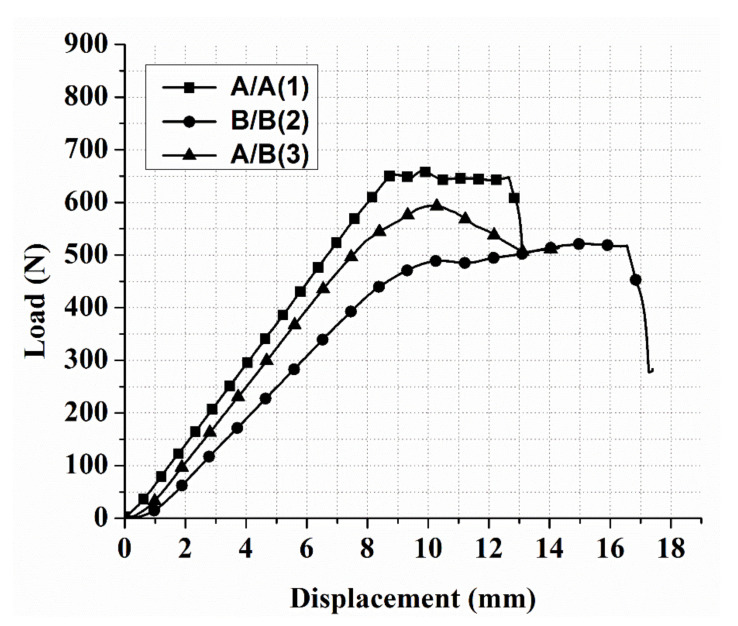
Load–displacement curves for CFRP composites with different stacking sequences.

**Figure 5:**
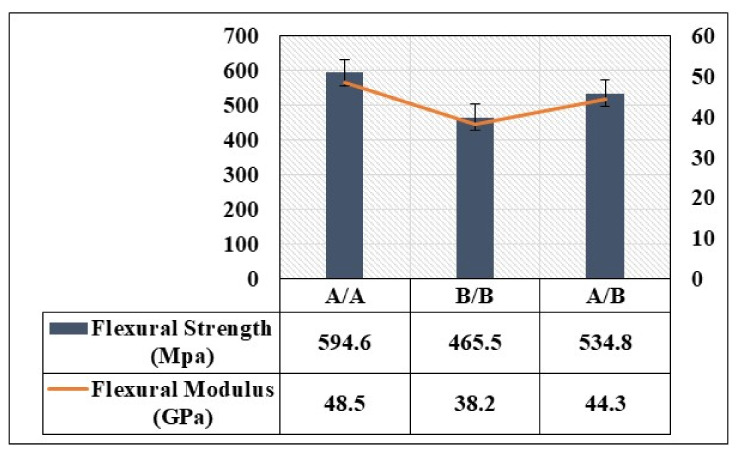
Flexural strength and flexural modulus of the composite laminate.

The load–displacement curves obtained for analysis of the flexural strength and modulus of the composite laminates are shown in Figure 4 and Figure 5. As can be seen in Figure 4, the performance of the flexural properties of the different stacking sequences was similar in terms of linearity up to peak load. Among all the stacking sequences, [A/A] exhibited the highest peak load, flexural strength, and flexural modulus due to the symmetry of the structure and balanced structure. The curve showed the lowest displacement among all the tested structures, revealing a brittle property. Following the peak load, the structure displayed cracks and delamination in the upper ply, indicting an occurrence of damage on the compressive side of the composite laminate. After the crack, the structure observed a plateau region, meaning there was no more effect on the surface of the composite. Subsequent to the plateau region, the structure reached a critical point in carrying a heavy load, leading to final failure. The [B/B] and [A/B] stacking sequences demonstrated linearity, ductility (indicating a high displacement due to the high elongation property of this structure), and plateau behavior. Figure 5 shows that the [B/B] stacking sequence had the lowest peak load, flexural strength, and modulus. This is because the orientation of the ply was a critical issue in the structure. The initial slope of the load–displacement curve of [B/B] played a vital role in the flexural strength and flexural modulus value. It was observed that the highest displacement curve had the lowest flexural strength and flexural modulus.

The ply-stacking sequence was found to influence the flexural modulus and flexural strength. For all the stacking sequences used in this study, the initial slope of the load–displacement curves exhibited non-linear characteristics due to initial damage that took place on the compressive side. The orientation of the layers in the composite laminate could improve the stability of the mechanical properties, which depended on the stacking sequence. The main reason for the difference between the three stacking sequences may be attributed to the fiber directions with respect to the line of action of applied bending force [37,38]. This can be seen when the stacking sequences with a large number of 90° changes in orientation had less flexural strength and flexural modulus. Moreover, the small variations in laminate thicknesses may have played a role in this distinction. During the flexural testing, it was observed that the laminates’ failure began from the outer layers and progressed to the inner layers. Thus, the greater the bending stiffness on the composite’s outer layers, the greater the flexural strength or force obtained. These observations can be valuable in analyzing the impact performance of composites with various stacking sequences.

### 3.2. Failure Modes under Flexural Loading

**Figure 6:**
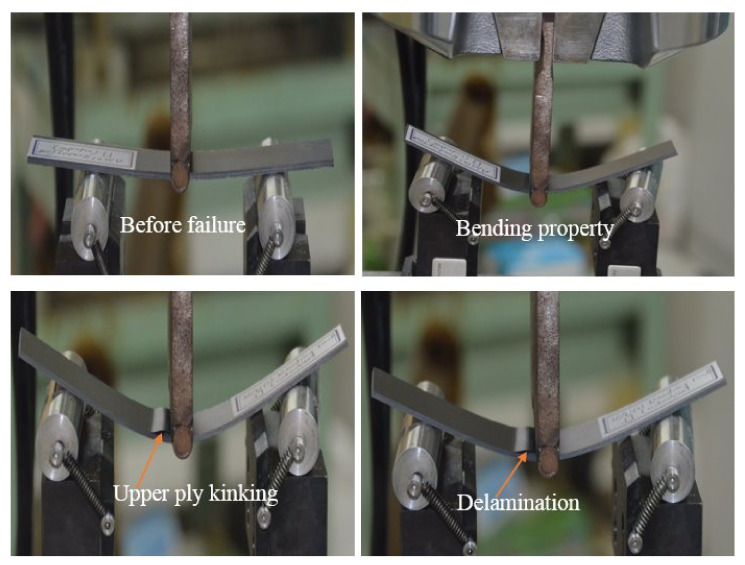
Visually inspect failure modes of the composite laminates.

**Figure 7:**
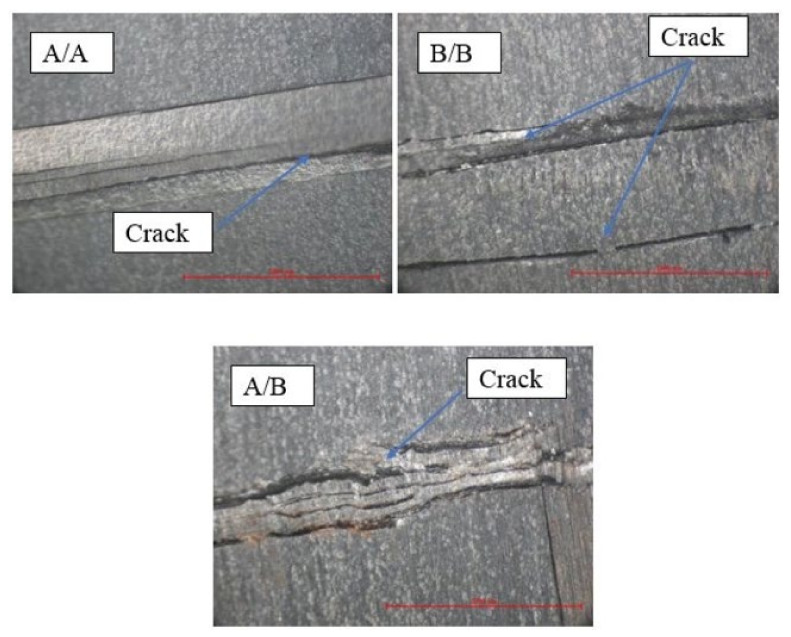
Optical images of failure surfaces (compressive side) of the composite laminates after flexural tests.

Common failures identified due to flexural loading included tensile failure, compressive failure, delamination, and shear. Nonetheless, failure by compression was found to be the most common [7]. Figure 6 depicts the visual inspection of the specimens following the flexural tests, with the failure modes observed being crack, upper ply kinking, and delamination. Hence, the failure modes were directly dependent on the laminate’s stacking sequences. Figure 7 shows optical images of the failed surfaces (compressive side) of the composite laminates after flexural tests. The shapes of the cracks that occurred on the compressive side of [A/A], [B/B], and [A/B] were different because of the orientation of the plies and stiffness properties.

### 3.3. Impact Properties

**Figure 8:**
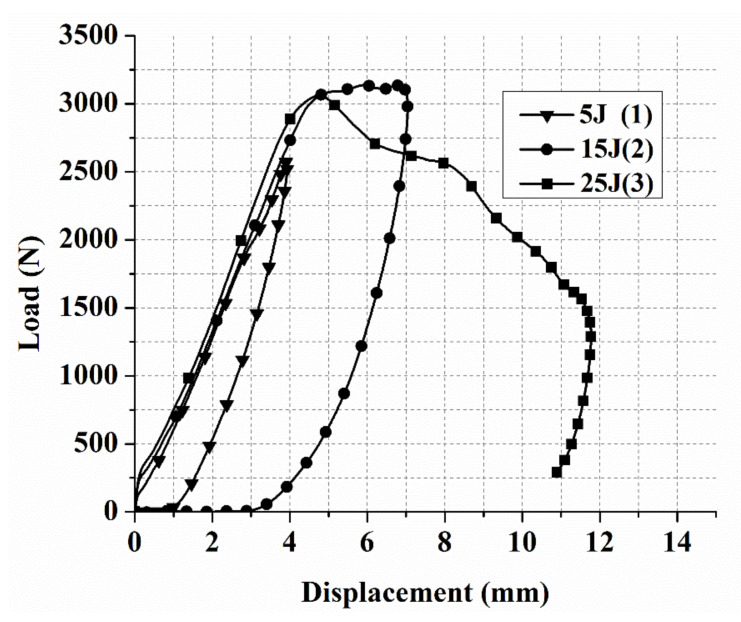
Load–displacement curves for low-velocity impact tests of laminate composites with [A/A] at 5, 15, and 25 J.

**Figure 9:**
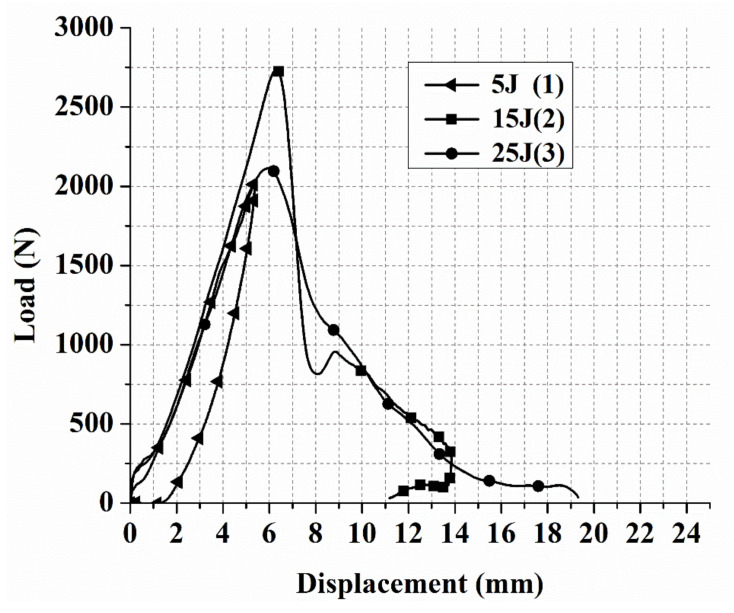
Load–displacement curves for low-velocity impact tests of laminate composites with [B/B] at 5, 15, and 25 J.

**Figure 10:**
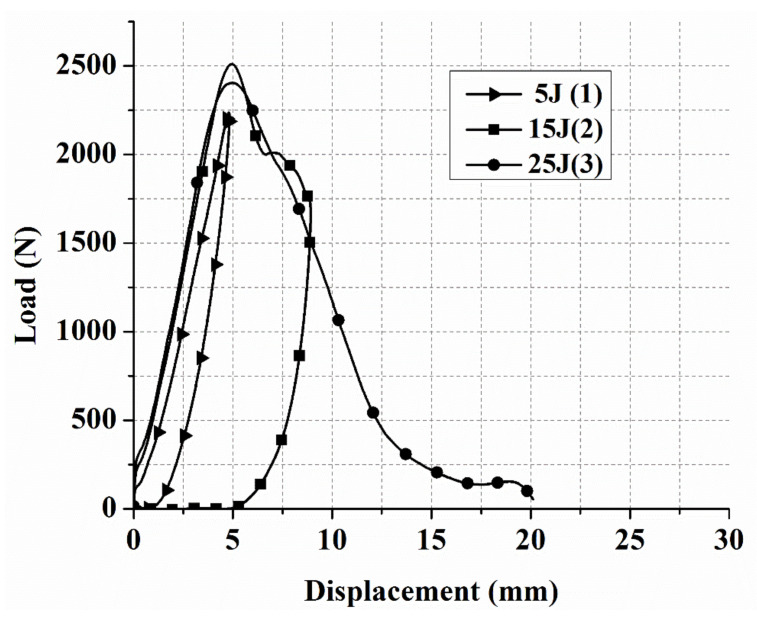
Load–displacement curves for low-velocity impact tests of laminate composites with [A/B] at 5, 15, and 25 J.

The laminates’ failure modes and impact damage were evaluated from the various obtained energy levels of load–displacement plots. The overall extent of damage on specimens after an impact event was assessed by visual, SEM, C-scan, and optical microscopy inspection. The impact damage failure modes varied with the impact energies on the laminate composite structures. The damage modes were characterized by combinations of matrix cracking, surface buckling, delamination, fiber pull-out, fiber fracture, and penetration. The impactor could perforate the impacted surface depending on the impact energy. Several tests were performed under three impact energies (5 J, 15 J, and 25 J) in order to examine the damage progress in the stacking sequences of [A/A] (Figure 8), [B/B] (Figure 9), and [A/B] (Figure 10).

Figure 8, Figure 9, Figure 10 illustrate the load–displacement plots of different composite laminate structures at 5, 15, and 25 J impact energy, respectively. At 5 J, the behavior of all stacking sequences can be explained by the structure in the first stage of bending stiffness having reached a peak load and rebounding to the original point in the graph. Because of the composite laminate’s resistance at 5 J and the absence of visible damage on the front and back surfaces, a closed-type curve was obtained. The absorbed energy during the impact event is represented by the area under the closed curve.

The response of all stacking sequences at 5 J in Figure 8, Figure 9, Figure 10 were found to have varied peak loads, displacement, and area under the curve (absorbed energy). These distinctions were attributed to the effect of stacking sequences, lay-up, and manufacturing conditions. The highest peak load and lowest displacement (indicating brittleness) were recorded in [A/A] for all the stacking sequences tested at 15 J. At 15 J, the first stage of all stacking sequences was similar to the 5 J case in terms of bending stiffness, i.e., at this point, the structures could carry loads. The maximum carrying load for the [A/A] structure was 3137.3 N (Figure 8). After this point, this structure could not have any additional load, and even a slight oscillation was observed to initiate damage at the compressive side of the composites.

The response of the [B/B] stacking sequence to impact damage at 15 J, as shown in Figure 9, was different from that of other structures. The curve showed a rapid and steep load rise at 15 J. After reaching a high load of 2735.8 N, the structure was unable to support any additional load, resulting in fiber breakage under the impact site, matrix crack, fiber pull-out in the back surface, and delamination. The type of curve obtained was an open curve at 15 J for [B/B], meaning that the specimens were either penetrated or perforated by the impactor during the impact. Figure 10 shows the failure modes of the [A/B] stacking sequence at 15 J after carrying a high load of 2510.5 N; fiber breakage in the upper and bottom surface, fiber pull-out in the back surface, matrix crack, and delamination were observed. Because the impactor made contact with the upper layer, the damage occurred without penetrating the specimen and returned to the original point in the graph, resulting in a closed-type curve. Because of the stability of the structure and the symmetry of their ply-stacking sequence, [A/A] (Figure 8) exhibited the highest peak load of all stacking sequences, at 15 J (quasi-isotropic stacking sequence). At 25 J for all the stacking sequences, the laminate response to impact damage was almost similar in terms of the curve type obtained (Figure 8, Figure 9, Figure 10). It was an open curve, which means that the damage occurred when the impactor penetrated or perforated the specimen during impact. The peak load at 25 J for the [A/A], [B/B], and [A/B] was 3058.2, 2113.7, and 2403.7, respectively.

For all types of composite laminate stacking sequences, if the damage was not visibly detectable because 5 J and impact indentation depth were less than a given value, this was referred to as barely visible impact damage (BVID). It occurs when blunt objects impact at low velocities; similar findings have been reported in earlier studies [39,40]. In the case of BVID, it is preferable to use non-destructive techniques to detect internal damage that cannot be visually inspected. Several studies have used C-scan [41,42], NDT techniques and numerical simulations [43], optical fiber sensors [40], FBG-based sensors [44], acoustic emission [45], and guided wave signals [46] to evaluate and detect BVID in composite laminates. Because of their perforation at 15 J, all the stacking sequences recorded the highest peak load among other levels of impact energy in the same laminate structure (Figure 8, Figure 9, Figure 10). These results suggest that the impactor did not cause further damage to the specimens even as the impact energy increased, and that the composite laminates reached a critical point for carrying high loads. The impact energy, displacement, and absorbed energy rose for all stacking sequences under different impact energy levels. In all composite laminates, an exponential increase in impact energy changed the curve type from closed type to open type.

### 3.4. Impact Damage Modes

#### 3.4.1. Visual Inspection

**Figure 11:**
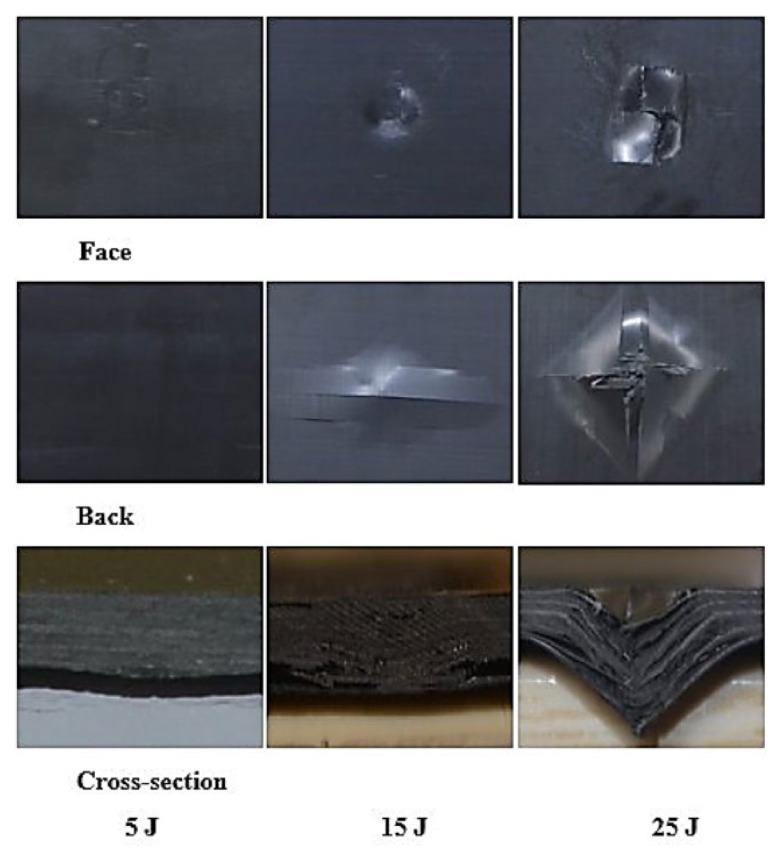
Pictures of the top and bottom surfaces and cross-sectional view of [A/A] at 5, 15, and 25 J.

**Figure 12:**
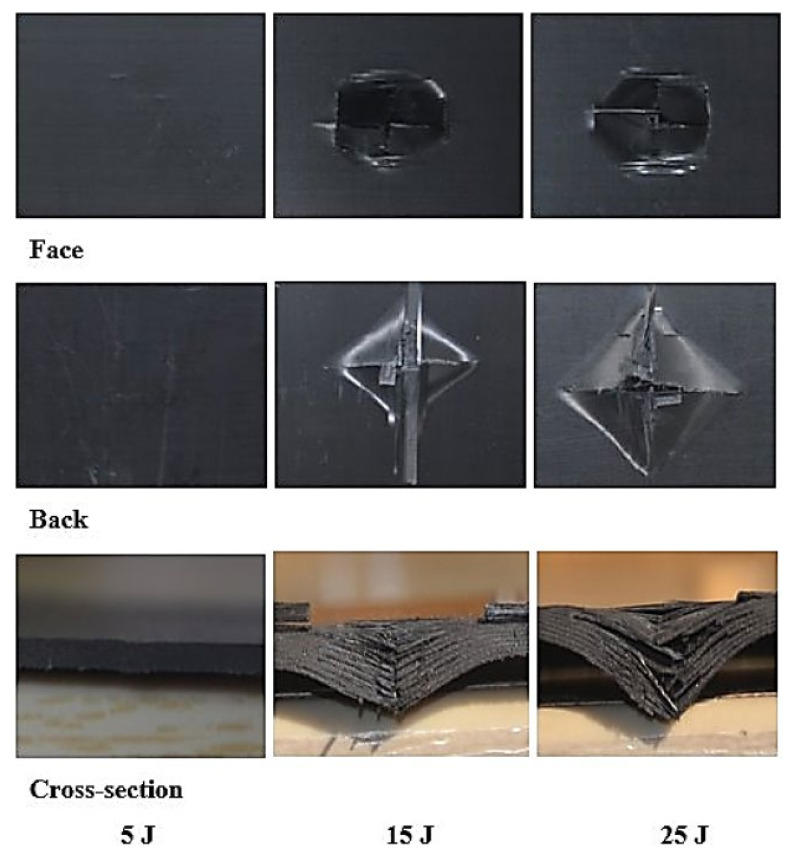
Pictures of the top and bottom surfaces and cross-sectional view of [B/B] at 5, 15, and 25 J.

**Figure 13:**
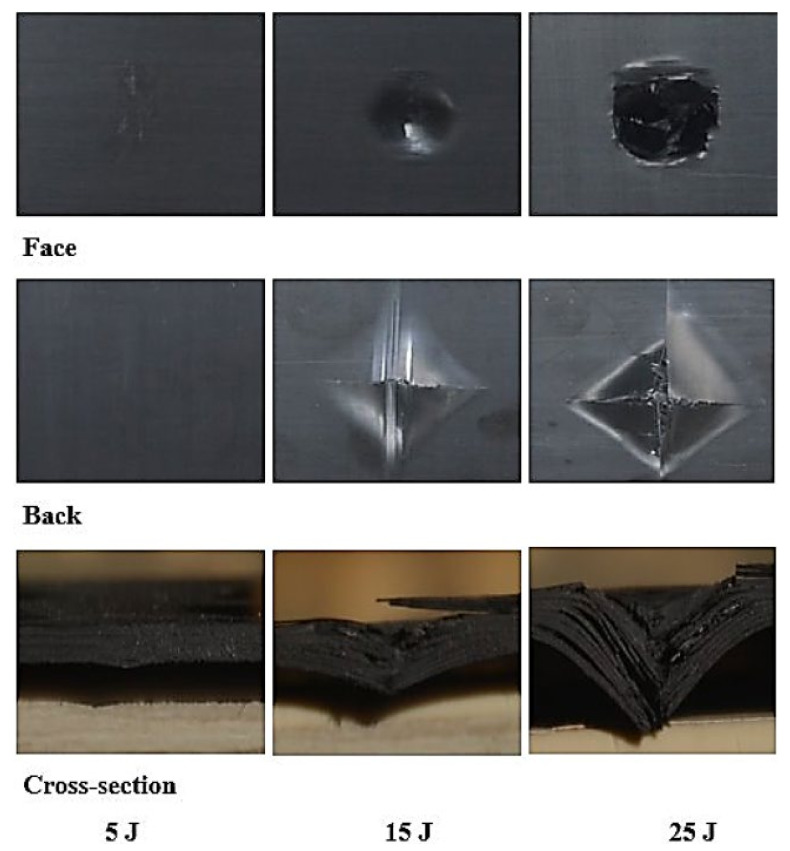
Pictures of the top and bottom surfaces and cross-sectional view of [A/B] at 5, 15, and 25 J.

Figure 11, Figure 12, Figure 13 show damage modes of [A/A], [B/B], and [A/B], respectively, and the damaged areas on the face, back surface, and the cross-section of the composite laminate after impact tests. The damage shapes and sizes in the delaminated areas varied depending on the materials’ stacking sequences, fabrication processes, and mechanical properties of ply. Upon visual inspection, no failure was observed in the face, back surfaces, or cross-section view at 5 J due to the structures’ resistance to impact damage (Figure 11, Figure 12, Figure 13). At 15 J, all the composite laminates were observed to be damaged, with failure in the face, back surfaces, and cross-section because of the degradation. According to the lay-up and critical point to carry high loads, the size and damage shape were different in the face, back surfaces, and cross-section. Damage areas were located in the center of the specimen due to the impactor being in contact with the sample’s surface at this point. The damage resistance was computed based on the final size and damage type in the specimen.

The penetration mode on [B/B] was observed to occur earlier at 15 J than the other two structures. The delamination and fiber breakage started to appear on the lower layers of [B/B], resulting in an earlier penetration of the impactor into the specimens. Visually inspected damage modes in the composite laminate included a circular shape on the facial surface, dent/depression, cross-sectional delamination, splits, and matrix crack and fiber breakage. At 25 J, the penetration mode was observed in the composite laminate since the fiber failure had reached a critical event, facilitating complete penetration of the specimens. The penetration process was profoundly influenced by orientation, fiber sizing, interface, weave architecture, and matrix type [47]. Several factors, such as laminate thickness, impactor mass, impact velocity, impact energy, geometry, ply thickness, striker tip geometry, stacking sequence, environment, and boundary conditions, determine how composite laminate is impacted by damage [30]. The damage size on the face surface of [A/A] was smaller than that of the other structures due to the quasi-isotropy and stability of this structure. The size of the damage increased with the rise in impact energy. The size, shape, and stacking sequence of the plate and the impact location significantly affected the impact deformation and damage formation. The degree of laminate orthotropy strongly affected the damage formation. Moreover, the clamp’s location, clamp geometry, and clamping force also affected the deformation of the specimen during impact.

#### 3.4.2. SEM Inspection

**Figure 14:**
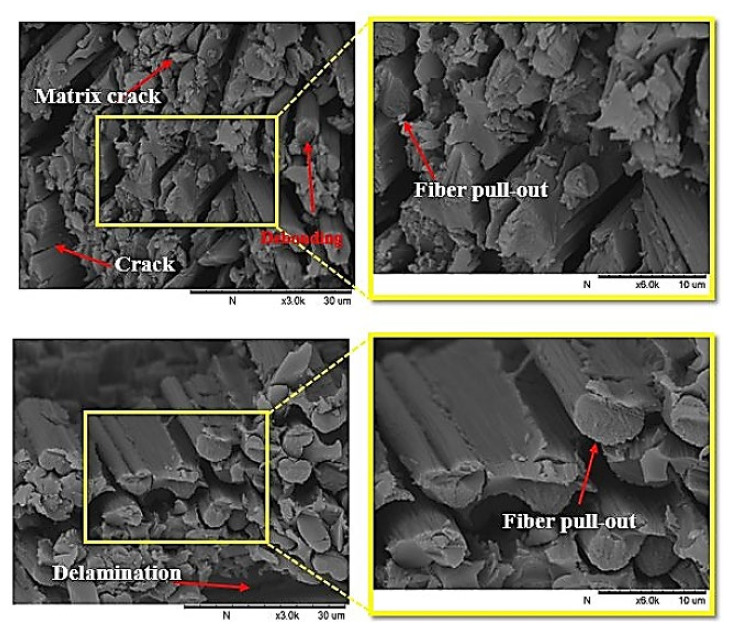
Low- and high-magnification SEM images of the cross-section of the fractured surfaces of the composite laminates at the face side after impact tests.

**Figure 15:**
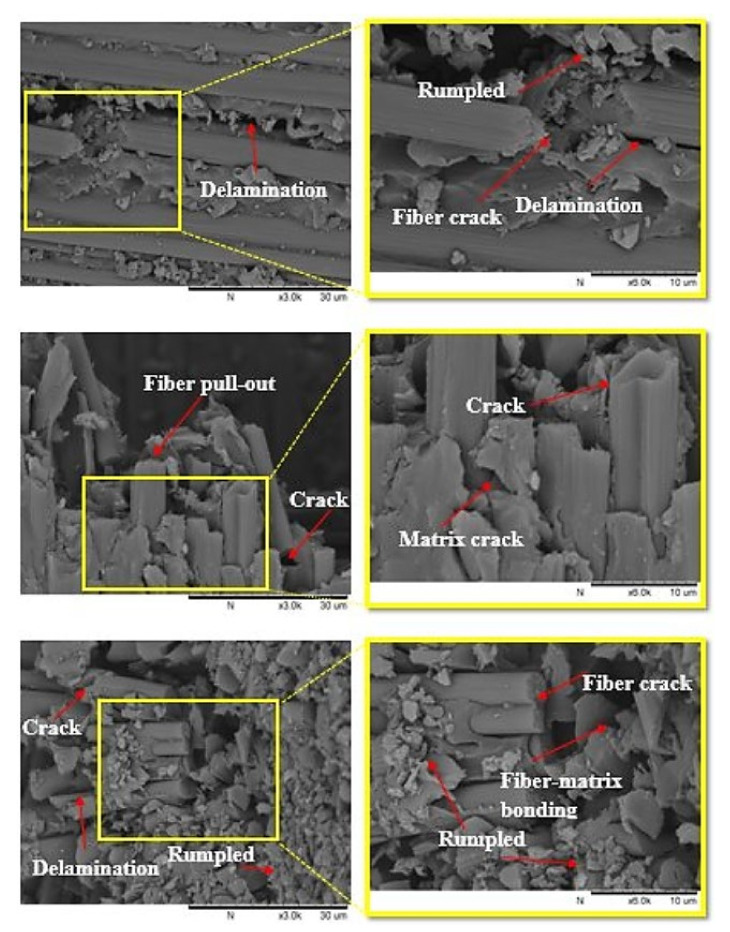
Low- and high-magnification SEM images of the fractured surfaces of the composite laminates at the face side after impact tests.

The damage behavior of the composite specimens was examined and analyzed using a scanning electron microscope (TM3000, HITACHI, Tokyo, Japan) after the impact tests. To conduct a thorough investigation, an inspection of the damage that occurred in the composite laminate was performed, and SEM was used to detect the crack in the surface and cross-section views of the composites. The SEM images of the specimens are shown in Figure 14 and Figure 15. The failure modes inspected by SEM were fiber pull-out, matrix/fiber debonding, delamination, and fiber crack. The failure occurrence can be explained first in terms of the transverse LVI-induced matrix crack. Matrix damage typically includes matrix cracking, fiber/matrix debonding, and delamination initiation. In the upper layers, the matrix cracks were initiated at the impactor’s contact edges. The global characteristics specific to the impacted specimens dictated the type of matrix cracking. The second failure occurrence was during the fracture process. This involved delamination and matrix cracking, which occurred first, followed by pull-out and fiber crack failure. High local stresses, non-impacted face (as a result of high bending stresses), and indentation effects (governed by shear forces) caused the fiber failure to occur below the striker. Delamination is defined as the process through which plies are separated in the resin-rich area. It is caused by bending stiffness variation between adjoining layers, that is, the contrasting fiber arrangement between the layers [47].

#### 3.4.3. C-Scan and Optical Microscopy Inspection

**Figure 16:**
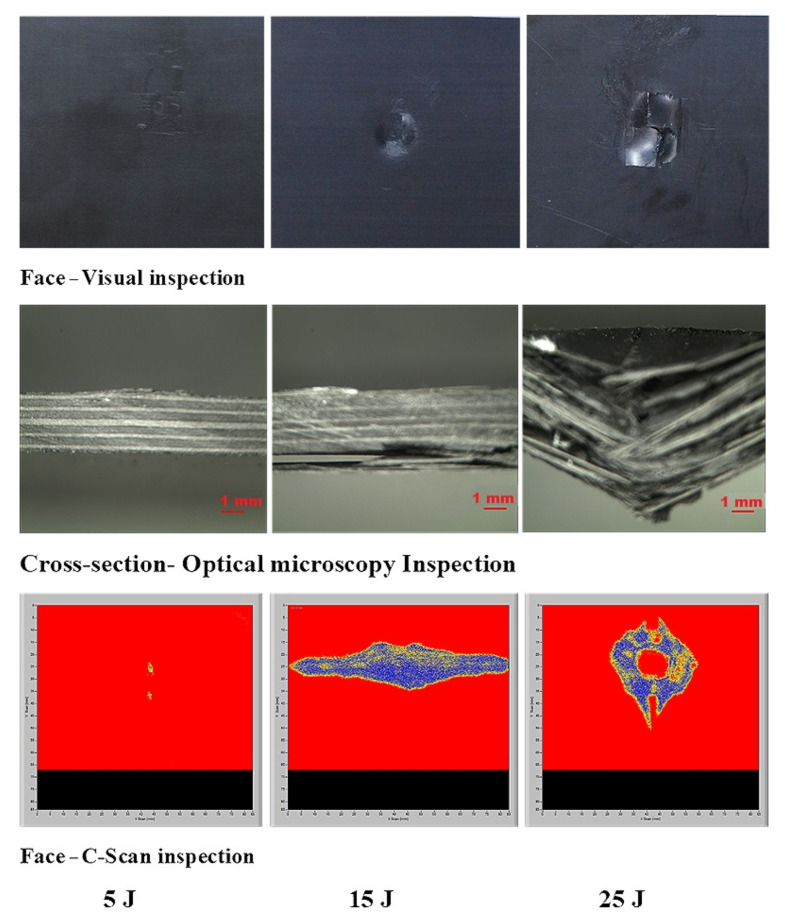
Pictures of the top surface view with visual, C-scan, and cross-section with optical microscopy of [A/A] at 5, 15, and 25 J.

**Figure 17:**
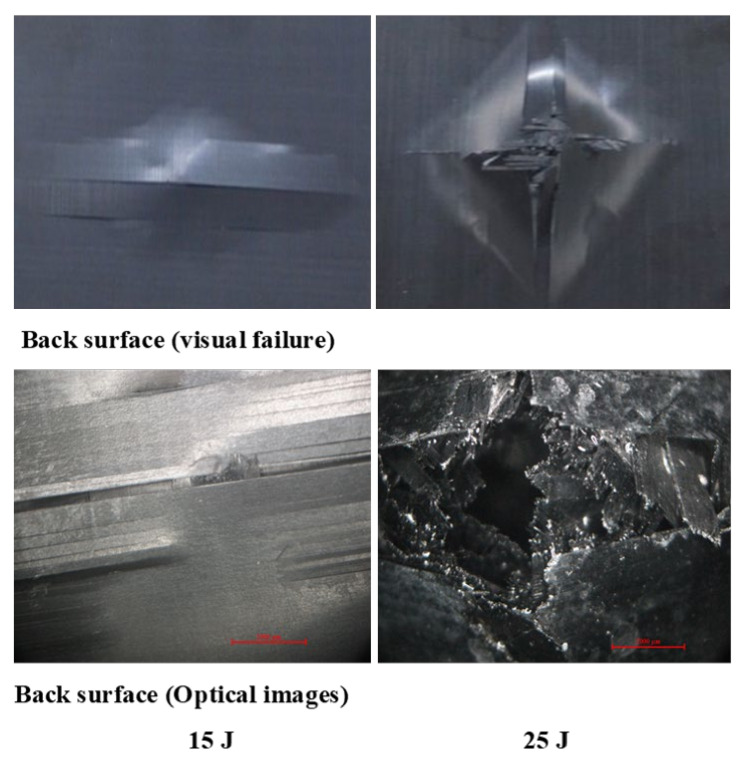
Pictures of the back surface failure view with visual and optical microscopy of [A/A] at 15 and 25 J.

**Figure 18:**
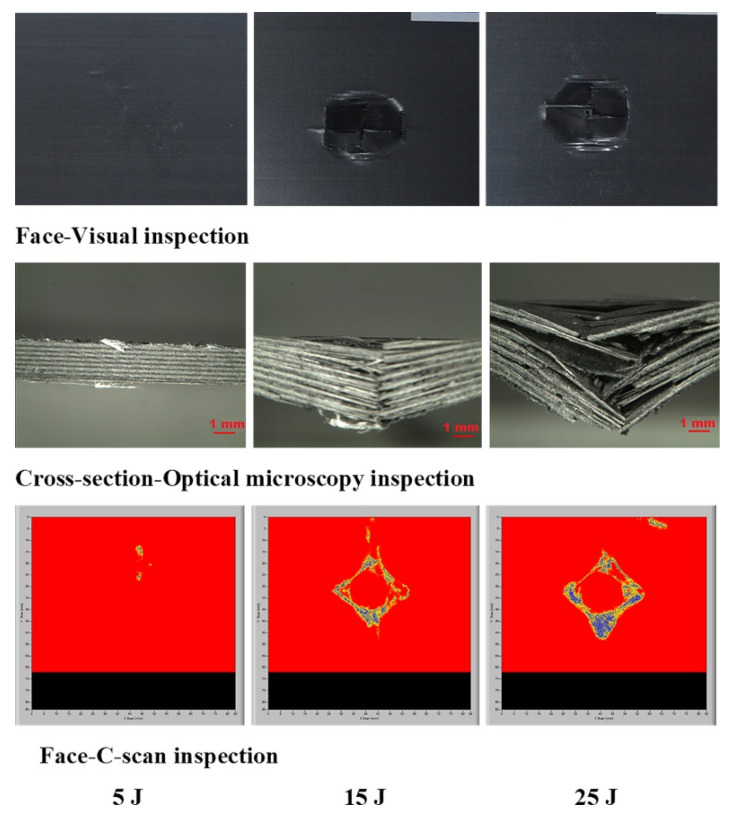
Pictures of the top surface view with C-scan and cross-section with optical microscopy of [B/B] at 5, 15, and 25 J.

**Figure 19:**
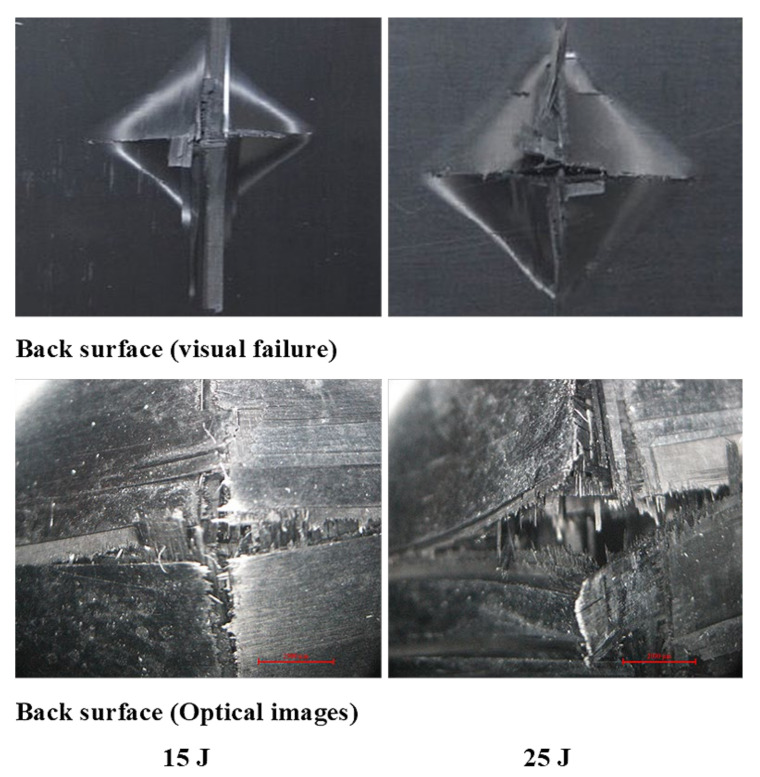
Pictures of the back surface failure view with visual and optical microscopy of [B/B] at 15 and 25 J.

**Figure 20:**
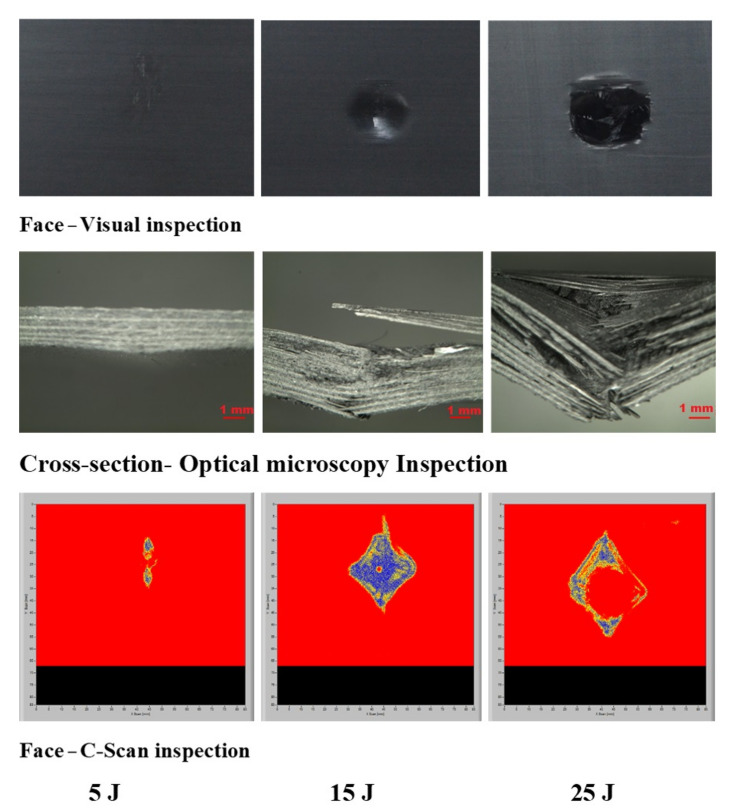
Pictures of the top surface view with C-scan and cross-section with optical microscopy of [A/B] at 5, 15, and 25 J.

**Figure 21:**
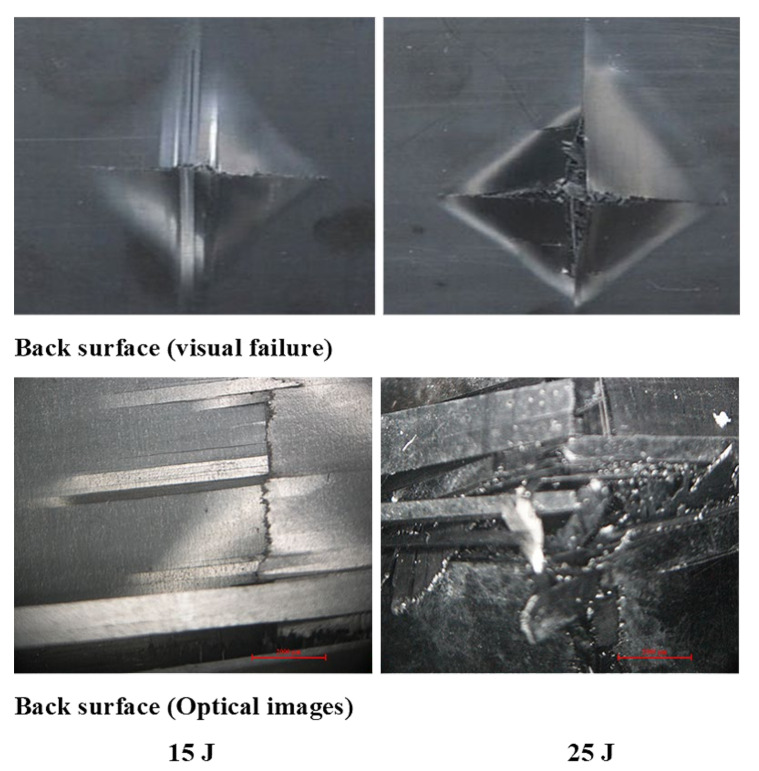
Pictures of the back surface failure view with visual and optical microscopy of [A/B] at 15 and 25 J.

In this study, air-coupled C-scan and optical microscopy images were obtained to study the possibility of determining the delaminated areas at the top surface, the size of the damage, and the bottom surface damage of the [A/A], [B/B], and [A/B] laminate composite structures (Figure 16, Figure 17, Figure 18, Figure 19, Figure 20, Figure 21). The internal inhomogeneity and damage scope were analyzed in C-scan mode. Varying the colors gave a detailed profile of the damage, elucidating the exact damage locations on both the top and bottom surfaces of the composite laminate. Figure 18 shows the comparison of the visual damage on the top surfaces at the cross-section area of the optical microscopy using the C-scan images of [A/A] at 5, 15, and 25 J, a convenient method. The results abstracted the final delaminated area by showing the measurement of the failure area that happened in the composite structures. The damage and C-scan image’s optical and visual shape illustrated that damage size increased with an increase in impact energy. Nonetheless, the composite material components’ properties and damage resistance capabilities gave the shape its non-static characteristic.

A delaminated C-scan image with blue and yellow colors showed the size and shape of the delamination in composite plates. When impact energy increased, the delamination area also increased, which could be attributed to the fiber orientation of the laminate composite structure. The size of the impact damage obtained from C-scan detection is always smaller than via visual inspection because the C-scan images only show the overlapping delamination area directly under the impact site. In contrast, visual inspection of the laminate surface and microscopic observations of the sample section illustrated the magnitude of the largest single delamination. The visual and C-scan inspection images at 15 J for [A/A] were not the same because C-scan combined the damages that occurred on the face and back surfaces. However, in the optical image of the cross-section at 15 J, the damage happened at the face surface of the sample along the fiber direction. Visual and optical microscopy back failure images of [A/A] at 15 and 25 J are presented in Figure 17. Failure images at the back surface are not shown at 5 J because no damage was detected by visual or optical inspection. The shape of the failure image on the backside was the same for both visual and optical microscopy inspection. The main damage modes were fiber breakage, fiber pull-out, splits/cracks, and indentation. Figure 18 shows that the damage shape by visual inspection was more apparent than the C-scan image and shows the same shape at the face side of the samples. The C-scan was manipulated to identify damage and delamination on the laminate’s backside caused by the distance between the pulse and receiver.

The size of the crack was found to be different between visual inspection and C-scan due to damage in the backsides caused by fiber shear-out. The final image of the damage was less visible than via visual inspection due to the shorter distance between the backside of the composite and the bottom probe (receiver). The optical cross-section failure and face C-scan failure images revealed that delamination occurred at 15 and 25 J, as seen in the C-scan with blue color and the fiber crack with yellow color (Figure 18). It was observed that 25 J brought about severe delamination. There was agreement between visual and optical microscopy damage modes on the back surface of the samples, as shown in Figure 19.

Deducing from Figure 20, the resulting area by C-scan exhibited only overlapping extended damage at every layer of the laminate composite structure. The extent of the damage impact at both 15 J and 25 J retrieved from the C-scan investigation was constantly smaller than via visual inspection. C-scan image was the most suitable way of detecting damage location sensitivity, damage size sensitivity, delamination, and surface distance. At 5 J, there was no visible damage in the face surface or optical microscopy cross-section image; however, a small failure was detected in the face surface by C-scan, which is a unique feature of C-scan. Delamination and fiber breakage were observed at 15 and 25 J in the optical cross-section and C-scan damage images. In addition, penetration was observed at 25 J due to the composite’s inability to bear more load, as it had already passed the critical point for carrying a heavy load. Figure 21 shows the failure shapes of visual and optical back surface images—almost the same damage modes, such as fiber breakage, fiber pull-out, and penetration, were evident at 25 J. The C-scan images illustrated minor delamination on the face surface of composite laminates at 5J for all stacking sequences (Figure 16, Figure 18, and Figure 20). However, the visual inspection did not reveal delamination because the C-scan modes only showed the extent of damage and internal inhomogeneity. At 15 and 25 J, the damage shape in both the visual and C-scan inspection was totally in agreement for [B/B] and [A/B], depending on the type of damage in the composites. The penetration depth in the 25 J case was more significant for all the structures than in the 15 J case because the composite material had reached the critical point in carrying any additional load at 25 J (Figure 16, Figure 17, Figure 18, Figure 19, Figure 20, Figure 21). A similar damage tendency was observed in composite materials when the impact energy was raised after reaching the crucial point for carrying a load. Since the penetration mode shape in the C-scan image was circular, the damaged part of the center was colored red. The size of the failure areas of the composite laminate obtained by C-scan are presented in Table 3.

### 3.5. Performance Index

A study was conducted to investigate the mechanical properties of three stacking sequences used in composite laminations of vehicle hoods. As asserted in [48,49], it is imperative to estimate the crash performance of the measured structure based on non-dimensional parameters, referred to as the performance index. Data collected from the impact test were used to determine the absorbing energy moment parameter (AEMP) using Equation (4) and the performance index (PI) with Equation (6). Various impact velocities (2.10, 3.63, and 4.69 m/s), equivalent to applied impact energies of 5 J, 15 J, and 25 J, respectively, were used to calculate the maximum energy absorbed by a structure during an impact load:

(4) $$AEMP=\frac{E_{max}}{M_{max}}$$

where

(5) $$M_{max}=\frac{P_{max}*a}{4}$$

The performance index:

(6) $$PI=\frac{AEMP}{RD^{2}}$$

where

(7) $$RD=\frac{Def_{max}}{a}$$

where a is the span length (mm) of the impact specimen, $E_{max}$ is the maximum absorbed energy (J), $M_{max}$ is the maximum moment in the panel corresponding to the maximum contact force, $P_{max}$ is the maximum contact force (N), and $Def_{max}$ is the maximum deformation (mm).

The parameter in Equations (4) and (6) shows the amount of energy absorbed by a structure due to non-dimensional deformation, which offers conclusive data about the panel’s crash performances. Table 4 presents the findings of the AEMP ratio, high peak load, PI value, and calculated absorbed energy. A high PI value was recorded in the [A/A] stacking sequence at 5 J, alluding that that composite laminate plate absorbed more energy without causing extreme deformation to the inner surface. This is one of the fundamental criteria of a crash-resistant structure that are required in the transportation industry. In another dimension, the [A/A] and [A/B] stacking sequences at 25 J recorded a high AEMP ratio, suggesting that a small load amount was moved to the support relative to the impact energy. Additionally, a high AEMP was observed at an impact energy of 25 J at a velocity of 4.69 m/s in both stacking sequences. The results suggest that with a high AEMP at a velocity of 4.69 m/s, there was a small transfer of energy to the base.

## 4. Conclusions

The current study looked into the flexural properties, low-velocity impact tests, and damage characterization using SEM, C-scan, and optical microscopy. The selection of proper stacking sequences can improve the crash performance features of automotive composite structures, making them more applicable to a wider range of applications. Based on the obtained results, the ply-stacking sequences were found to influence the flexural behavior of tested structures. For instance, the highest load, flexural strength, and flexural modulus were exhibited by the laminate with a stacking sequence of [[0, 90, 45, −45]_2_, 0, 90]_S_. The failure modes of the composite laminate depended on the maximum bending stiffness and the ability of the individual constituent material to carry loads. According to the impact data, each of the laminates demonstrated a high peak load below an impact energy of 15 J. Under 15 J impaction, [[0, 90, 45, −45]_2_, 0, 90]_S_ showed the highest peak load and lowest displacement (indicating brittleness). The size of the impact damage obtained from C-scan detection is always smaller in comparison to visual inspection, as C-scan images only show overlapping delamination areas directly under the impact site. Moreover, a high AEMP ratio was observed in the [[0, 90, 45, −45]_2_, 0, 90]_S_ and [[0, 90, 45, −45]_2_, [0, 90]_6_] stacking sequences at 25 J, indicating a lower amount of load transfer to the supports than impact energy. However, a high PI value was observed in the [[0, 90, 45, −45]_2_, 0, 90]_S_ stacking sequence at 5 J, suggesting that the composite laminate plate was able to absorb a high amount of energy without transferring excessive deformation to the inner structure, which is one of the main requirements of a crash-resistant structure for transportation. The fabrication of a composite laminate structure for automobile parts that is enhanced by different stacking sequences to reach the maximum possible impact energy, as well as flexible properties during a crash, is deferred to future work. The design of composite laminates capable of satisfying impact and crash performances would be quite interesting for automobile industries.

## Figures and Tables

**Table 1:** Properties of the UD carbon fiber/epoxy prepreg.

Property	Value
Fiber areal weight (g/m^2^)	125
Resin content (%)	30
Prepreg total weight (g/m^2^)	179
Thickness (mm)	0.13

**Table 2:** The specifications of the stacking sequences.

Symbol of Stacking Sequence	Lay-Up	Number of Layers	Thickness (mm)
[A/A]	[[0, 90, 45, −45]_2_, 0, 90]_S_	20	2.41
[B/B]	[0, 90]_10_	20	2.43
[A/B]	[[0, 90, 45, −45]_2_, [0, 90]_6_]	20	2.45

**Table 3:** Measurements of the fail areas for the composite laminates.

Lay-Up	5 J	15 J	25 J
**[A/A]**	4.84 mm^2^	491 mm^2^	423.3 mm^2^
**[B/B]**	7.96 mm^2^	117.2 mm^2^	179.6 mm^2^
**[A/B]**	33.16 mm^2^	318.64 mm^2^	233.6 mm^2^

**Table 4:** Summary of the results of non-dimensional parameters.

Lay-Up	Velocity (m/s)	Impact Energy (J)	Absorbed Energy (J)	High Peak Load (N)	AEMP	PI
**A/A**	2.10	5	5.02	2571.4	0.000073	0.047
	3.63	15	15.002	3137.3	0.000013	0.0026
	4.69	25	25.04	3058.2	0.00033	0.023
**B/B**	2.10	5	5.019	2012.6	0.00001	0.0034
	3.63	15	15.01	2735.8	0.00022	0.011
	4.69	25	14.9	2113.7	0.00028	0.00013
**A/B**	2.10 3.63 4.69	5 15 25	5.01 15.0018 20.4	2235.19 2510.5 2403.7	0.00009 0.00023 0.00033	0.038 0.029 0.00031

## Data Availability

Data are contained within the article.
